# A novel logical model of COVID-19 intracellular infection to support therapies development

**DOI:** 10.1371/journal.pcbi.1010443

**Published:** 2022-08-29

**Authors:** Elena Piretto, Gianluca Selvaggio, Damiano Bragantini, Enrico Domenici, Luca Marchetti

**Affiliations:** 1 European Institute of Oncology IRCCS, Milan, Italy; 2 Fondazione The Microsoft Research—University of Trento Centre for Computational and Systems Biology (COSBI), Rovereto, Trento, Italy; 3 Infectious Diseases Unit, Pederzoli Hospital, Peschiera del Garda, Italy; 4 Department of Cellular, Computational and Integrative Biology (CIBIO), University of Trento, Povo, Trento, Italy; University Hospital RWTH Aachen, GERMANY

## Abstract

In this paper, a logical-based mathematical model of the cellular pathways involved in the COVID-19 infection has been developed to study various drug treatments (single or in combination), in different illness scenarios, providing insights into their mechanisms of action. Drug simulations suggest that the effects of single drugs are limited, or depending on the scenario counterproductive, whereas better results appear combining different treatments. Specifically, the combination of the anti-inflammatory Baricitinib and the anti-viral Remdesivir showed significant benefits while a stronger efficacy emerged from the triple combination of Baricitinib, Remdesivir, and the corticosteroid Dexamethasone. Together with a sensitivity analysis, we performed an analysis of the mechanisms of the drugs to reveal their impact on molecular pathways.

## Author summary

The paper introduces a logical-based mathematical model of the cellular pathways involved in the COVID-19 infection. The aim of the model is to study, in a qualitative but comprehensive way, the cellular mechanisms developed during the virus infection with the principal focus on drug treatments. The model is able to reproduce various illness scenarios: from the early infection stages to the late illness stages characterized by strong immune reaction usually evolving in the so-called cytokine storm. Different drug effects have been tested singularly and in combination treatments. Computational sensitivity analysis was performed on the model along with the analysis of the mechanisms of the drugs to reveal their impact on molecular pathways. The results show that the effect of single drugs may be limited or counterproductive, depending on the illness stage. The highest predicted efficacy is obtained by combining three different treatments: the anti-inflammatory Baricitinib, the anti-viral Remdesivir and the corticosteroid Dexamethasone. This triple combination therapy has been analyzed not only in terms of global cellular effect but also in function of the involved internal pathways, suggesting the rational mechanisms for its successfulness.

## Introduction

In this century, the human population faced several epidemic cycles of pathogenic coronaviruses: the severe acute respiratory syndrome coronavirus (SARS-CoV) in 2002–2003 [1], the Middle East respiratory syndrome coronavirus (MERS-CoV) in 2012 [2,3] and, the ongoing severe acute respiratory syndrome coronavirus 2 (SARS-CoV2) causing the Coronavirus Disease (COVID-19) [4]. The World Health Organization (WHO) declared the SARS-CoV-2 (COVID-19) outbreak a global pandemic on March 11th, 2020.

According to WHO, from 2019, there have been hundreds of millions of confirmed cases of COVID-19 with millions of deaths all over the world. The national healthcare systems have been under strong pressure, restrictions on the movement of people imposed, strict health measures introduced, schools closed, and the world economy experienced a sharp slowdown [5]. At the beginning of 2021, different vaccines have been approved by the U.S. Food and Drug Administration (FDA), by the European Medicines Agency (EMA) [6], and other regulatory agencies. However, the rapid mutation rate of the virus, together with the relatively slow process of mass vaccination, caused by people’s distrust and the disparity in distribution of the vaccines among countries, hampered the pandemic’s end [5]. Despite the rapidity with which several effective and safe vaccines have been developed, only mild improvements have been obtained in terms of medication for treating the disease.

From a clinical point of view, COVID-19 is characterized by a high rate of contagiousness but with a relatively small probability of developing a severe form of the disease. About 20% of the infected subjects develop clinically relevant symptoms, while the majority of the subjects remain asymptomatic or pauci-symptomatic [7]. These percentages are reshaped according to risk factors such as age, diabetes, obesity, or heart disease [8].

At the cellular level, the disease starts when the virus first binds the upper airway epithelial cells (mainly through the Spike protein (S) and the angiotensin-converting enzyme 2 (ACE2) receptor), it then enters the cells and begins to replicate [7]. Typically, the innate immune system is stimulated by the viral replication and eventually triggers the adaptive response that eradicates the virus [9]. However, the reduced innate antiviral response, i.e., low level of type I and III interferons, combined with the sustained expression of pro-inflammatory cytokines (due to nuclear factor kB (NF-kB)) lead to a hyperstimulation of the immune system and eventually to respiratory distress [10,11].

The observed symptoms, together with the biological knowledge, lead to hypothesize a biphasic behavior of the disease characterized by a first phase of viral replication with mild symptoms and a second phase with severe symptoms and a dysregulated immune system with acute levels of inflammation [12]. Therefore, it is crucial to consider the biphasic nature of the disease to accordingly plan the treatments, by administering the optimal combination therapies.

Currently, the only approved small molecule drug by the FDA for COVID-19 is Veklury (Remdesivir) (*i*.*e*., an antiviral drug approved for patients requiring hospitalization). To rapidly identify new therapies for COVID-19, numerous repurposing studies aimed at finding alternative uses for previously approved drugs have been carried out both *in-vitro* [13] and *in-silico*, and with different techniques [14,15], including artificial intelligence [16]. At present, some pharmaceuticals have been authorized for emergency use against the coronavirus, and some protocols are in various phases of clinical trials [17].

Mathematical models have been developed to study the pandemic evolution of SARS-CoV2 [18]. The techniques used to study the infectious disease dynamics [19] are mainly: statistical-based methods for epidemic surveillance (*e*.*g*., regression techniques), mechanistic state-space models (*e*.*g*., agent-based, SIR models) and, machine learning based models (*e*.*g*., based on data mining).

Fewer mathematical models have instead been developed to study COVID-19 disease in a mechanistic way. Different scales of investigation and different mathematical tools have been used: genomic scale (mainly with artificial intelligence) [20,21], cellular-molecular scale and organs-systemic scale (with hierarchical-deterministic models) [22]. At the molecular level, most of the mathematical models focused on the immune system activation [23] and cellular signaling [24] using ordinary differential equation (ODE) models.

The scientific community has made a constant and progressive effort to build knowledge repositories to support the fight against the disease [25]. In particular, the COVID-19 Disease Map initiative [26] built molecular diagrams of virus-host interaction mechanisms addressing some of the known COVID-19 hallmarks (*e*.*g*., NLRP3 inflammasome activation, coagulation pathway, etc.). Part of this initiative is the curated causal relationship repository SIGNOR [27], which, using evidence from published articles or derived from analogous viruses (*i*.*e*., SARS, MERS), built a binary causative network that can be of great support in building logical models.

The purpose of this work is to develop a logical-based mathematical model of the molecular interactions inside an epithelial cell starting from the SIGNOR relationship repository. The model aims to reproduce the main cellular pathways involved in the COVID-19 infection to test different drugs (single or in combination), providing insights on their mechanisms of action.

## Results

### Model structure

**Fig 1:**
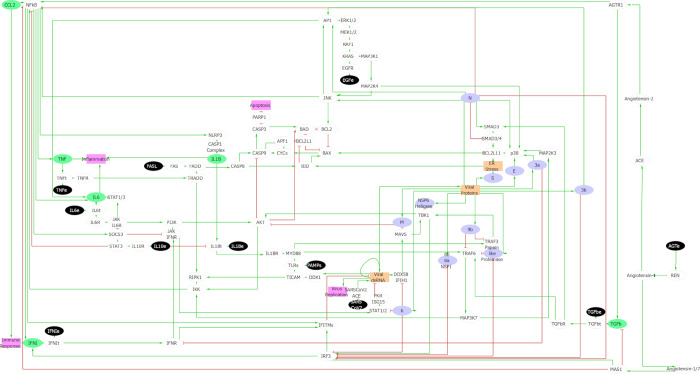
Regulatory network of COVID-19 infection. The response, controlled by the microenvironment, affects the cellular phenotypes through key molecular pathways. Inputs from the microenvironment are denoted in black, viral proteins are marked in violet, and cytokines in green. The phenotypic readouts Viral replication, Inflammation, Apoptosis and Immune response are indicated in pink. Inhibitions are denoted by red blunt arrows and activations by green arrows. Ellipsoidal components are associated with Boolean levels, whereas rectangles indicate multivalued level components.

We built a parsimonious regulatory network of the mechanisms involved in the viral-host interaction following the COVID-19 infection (**Fig 1**). The network was developed based on the curated binary relationships annotated in the repository SIGNOR [27]. We retrieved the cellular processes involved from the SIGNOR COVID hallmarks sections. Since our interest lies in viral reproduction and spread of the infection eventually leading to chronic inflammation and necrosis of the tissue [12], we decided to focus on virus entry, inflammation (*i*.*e*., cytokine storm or immune response), and apoptosis. SIGNOR repository is part of the COVID-19 Disease map project [26] and participates in mapping the interactions relevant for the COVID-19 pathology according to the current knowledge. When specific knowledge is lacking, indirect relationships and information derived from similar viruses (*i*.*e*., *SARS* and MERS) are included.

Afterwards, we built a model, using logical formalism, because it allows handling such complex networks even when there is a lack of quantitative data or detailed information for most regulatory mechanisms [28]. Logical models are composed of an influence graph where the nodes represent the system variables (*e*.*g*., genes, proteins, or phenomenological properties), and signed edges are the regulatory interactions (activation or inhibition). Each component is associated with a discrete variable (Boolean or multivalued), whose value represents the component functional level (*i*.*e*., level of expression, activity, complex formation, phenotypic state, etc.). Every component is then associated with a logical rule that defines the component values depending on the states of the regulatory components (*i*.*e*., their regulators). In our model, the logical rule of each internal component was defined based on experimental evidence when available. If not specified otherwise, a component activation requires the presence of at least one of its activators combined with the absence of all its inhibitors (**S2 and S3 Files**).

Alongside the input coding for the virus presence (SARS_CoV_2), we considered a series of environmental inputs such as the cytokines: IL-1β, IFN-I, IL-6, TNF-α, IL-10 and TGF-β, the epidermal growth factor (EGF), the peptide hormone angiotensin (AGT), the death ligand FASL and the pathogen-associated molecular patterns (PAMPs). All inputs and internal nodes are Boolean; that is, their levels convey the presence (1) or absence (0) of these signals in the microenvironment.

Four phenotypic readout nodes define the cell state: Infected, Apoptosis, Inflammation, and Immune response. The former two are Boolean variables, while Inflammation and Immune response are multivalued variables with maximum values respectively to 3 and 2. These values are a function of the number of cytokines secreted by the cell, thus identifying different degrees of inflammation or innate immune response (for more information on the logical rules, see **S3 File**).

The Inflammation readout is activated by the secretion of IL-6, TNF-α and IL-1β, whereas the immune system by the secretion of CCL2 and IFN-I. Many more cytokines and chemokines are involved in inflammatory processes or in immune system activation, however only few of them are over-produced in SARS. Among others, IL-6 and TNF-α are over-expressed in COVID-19 [22,29,30] while the IL-1 family is strongly associated with acute inflammation [31–33]. The rapid replication of SARS-CoV-2 induces the delayed release of IFN-α/β, which is accompanied by the influx of many pathogenic inflammatory mononuclear macrophages. The accumulated mononuclear macrophages receive activating signals through the IFN-α/β receptors on their surface and produce more monocyte chemoattractants (such as CCL2, CCL7, and CCL12), resulting in further accumulation of mononuclear macrophages. These mononuclear macrophages produce elevated levels of proinflammatory cytokines (TNF-α, IL-6, IL-1β, and inducible nitric oxide synthase), thereby increasing the severity of the disease [34]. Therefore, the model discriminates between the first immune response and the secondary adaptive response associated with the inflammatory process. With regards to the innate immune response, the model accounts for IFN-I and CCL2 among the most important players.

### Phenotypic repertoire and infection scenarios

The model dynamics can be described through a state transition graph (STG), where the nodes of the graph represent the model states, as an arrays of variable values, and the edges identify state transitions. The transition between two states of the STG is called by a change in value of a model variable according to its logical rule [28]. Depending on the updating scheme, competing variable updates can be treated differently. In this work, we used an asynchronous updating scheme, meaning that in the STG any state has as many successors as the number of variables that are called to update, leading to non-deterministic dynamics [28]. We did not make assumptions on the firing probability of the different model reactions to explore the whole STG and study all possible states. Given the discrete finite nature of the STG, a logical model simulation will always be asymptotically trapped either in a single stable state or several interconnected states (complex attractor).

As previously discussed, the model has ten Boolean environmental inputs (for a total of 2^10^ = 1024 combinations). The stable states identified are 4,630 (**S4 File**), suggesting the presence of multistability for some input combinations.

We inventoried the phenotypic repertoire that the model is producing, by listing all the phenotypes that appear at least once (**Table 1**), with the relative abundance to the total of stable states (**Table 1**- column percentage).

**Table 1:** Model phenotype repertoire with their relative abundance. The stable states have been grouped in six phenotypes: Viral (V), Apoptotic (A), Inflammatory Low or Medium or High (IL, IM, IH), Healthy (H). The states with null probability cannot be reached in untreated condition but will appear during treatments.

Phenotype	Infected	Apoptosis	Immune response	Inflammation	Percentage (%)	
**H**	0	0	0	0	0.54	0.54
**IL**	0	0	0	1	0.52	12.96
	0	0	1	0	6.22	
	0	0	1	1	6.22	
	0	0	2	1	0	
**IM**	0	0	2	2	0	0
**IH**	0	0	2	3	22.1	22.1
**A**	0	1	0	0	1.84	53.29
	0	1	0	1	2.07	
	0	1	1	0	2.42	
	0	1	1	1	2.76	
	1	1	0	1	44.2	
	0	1	2	2	0	
**V**	1	0	0	1	11.1	11.1
	1	0	0	0	0	

The 4630 stable states were mapped into 6 phenotypes according to the output nodes values. The Healthy phenotype (H), a Low Inflammation (IL), a Medium Inflammation (IM), and a High Inflammation (IH) phenotype, depending on the activation state of the two readouts Immune response and Inflammation, as outlined in **Table 1**. In particular IL corresponds to the activation of one of the internal pro-inflammatory cytokines (TNF-α, IL-6, IL-1β), IM to the activation of two and IH to the activation of all these three cytokines. The Healthy phenotype (H) can be obtained by setting all the inputs to zero and “no virus”, and leaving the system to evolve until its “physiological level”, characterized by all null readouts. However, the basin of attraction of the H phenotype is wider and can also be reached by particular input configurations (i.e., IFN-I and IL-10 external inputs active from time zero) corresponding to particular cases in which the cell receives these two signals from the boundary cells. From this point on, we will refer to the H state as the phenotype achieved with null inputs. The Viral phenotype (V) identifies a state where the virus has entered the cell and is actively replicating and, finally, the Apoptotic phenotype (A) defines a cell with active apoptotic process. Due to the irreversibility of the cell death machinery and the importance of the apoptotic signal for the elimination of infected cells, dominance of this signal over all the others has been assigned.

**Fig 2:**
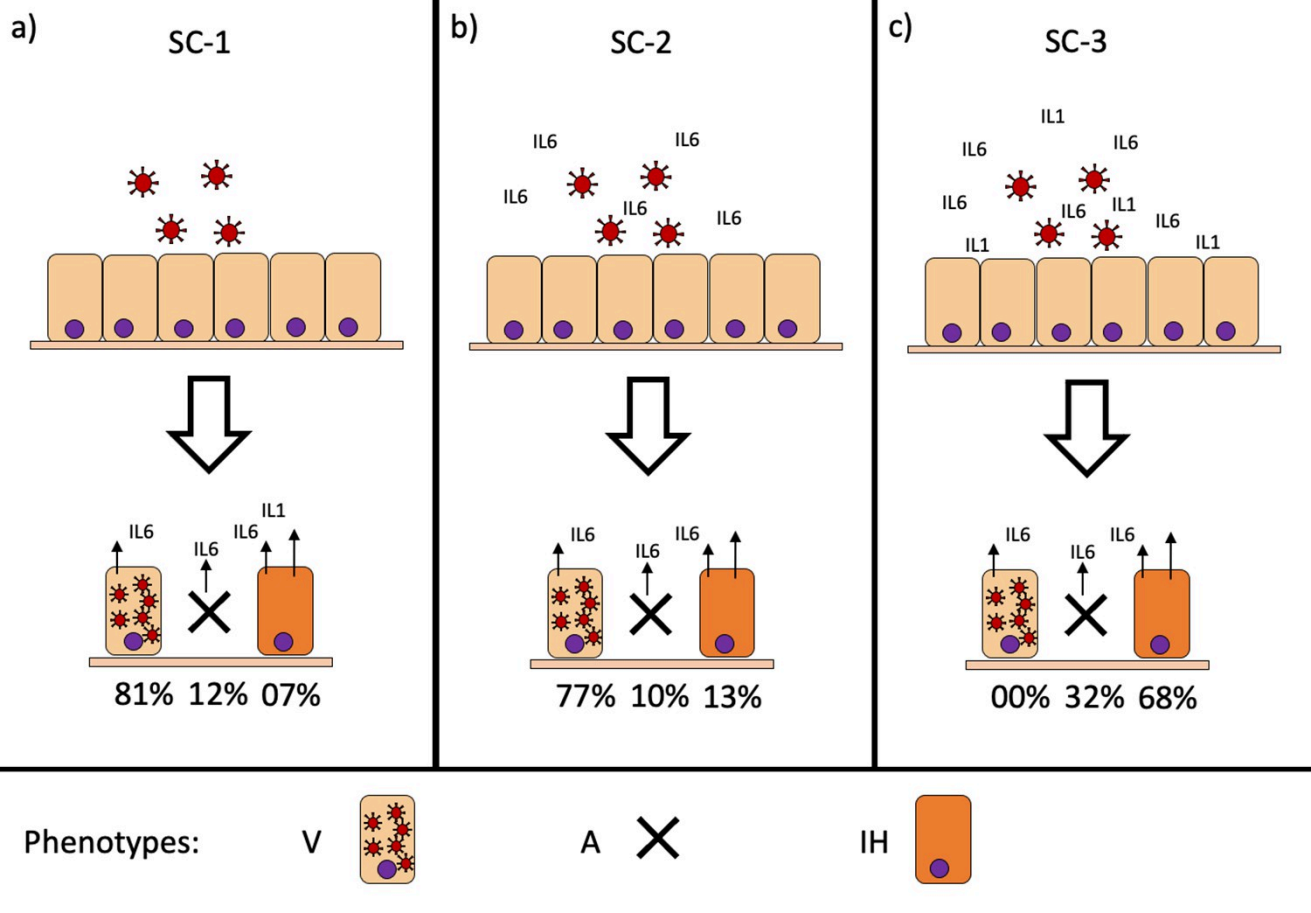
Infection scenarios. A healthy epithelium is exposed to the virus in three different microenvironmental conditions (SC-1, SC-2 and SC-3). Accordingly, to the extracellular cues, healthy cells can evolve into three different phenotypes: viral (V), apoptotic (A), inflammation high (IH) with the relative probabilities reported below (obtained using the GINsim functionality Avatar [36]).

As reported in the literature, COVID-19 has a biphasic behavior [8,12,35]; the initial phase is defined by the establishment of the disease with the infection of healthy cells, the following stage of viral replication causes localized inflammation in the lung, which eventually evolves into a systemic extrapulmonary hyperinflammation. We thus decided to study the dynamic behavior of the model (using the GINsim functionality Avatar [36], see Material and Methods) starting from an H initial condition and exposing the cell to different environmental stimuli according to the disease stage. The H state was obtained by setting all inputs to 0 and letting the model evolve to a stable state. We defined scenario 1 (SC-1), **Fig 2A**, an early infection of a healthy epithelium. By fixing SARS_CoV_2 to 1, and leaving all the others at 0, the model will evolve towards three possible phenotypes with different probabilities: V (81%), A (12%), and IH (7%). The V phenotype, dominant in SC-1, represents cells that have been infected and are actively producing viral protein to be assembled and released as a virion. The cell is also secreting IL6 as part of the inflammatory response. The A phenotype accounts instead for those cells that, once infected, reacted to the exogenous genetic material or viral protein by activating the apoptotic cascades. The IH phenotype instead identifies those cells that have been infected, but that managed through the innate response to overcome the virus, several cytokines (IL-6, IL-1β, TNF-α, IFN-I) together with the recruitment on site of the immune system (CCL2) generates a robust inflammatory response.

The secretion of pro-inflammatory cytokines in SC-1 phenotypes prompted us to evaluate how the virus would infect a healthy epithelium when the external input such as IL-6 (**Fig 2B**) or IL-6, TNF-α, IL-1β and IFN-I (**Fig 2C**) are activated; we called these two conditions scenario 2 (SC-2) and 3 (SC-3), respectively.

SC-2 identifies a mildly inflamed region; the endpoint phenotypes obtained are as before: V (77%), A (10%), and IH (13%), but with IH becoming dominant over A. The third scenario (**Fig 2C**), with IL-6, IL-1β and IFN-I, identifies a portion of the tissue with an ongoing cytokine storm (or strong inflammatory response). In this case, the stable phenotypes are dead cells (A phenotype 32%) and inflamed tissue (IH phenotype 68%).

Since the presence of local signaling molecules, such as cytokines, is an important factor in selecting the simulation scenario, we decided to investigate how a heterogeneous population of cells would react to the infection and what is the dynamic behavior of the system (simulations were performed with MaBoSS [37], see Material and Methods). We defined a family of simulations for each scenario by initializing the system to phenotypes H, V or IH (not A, since apoptosis is a terminal state) with different probabilities. To generate a heterogeneous initial state, we first selected the probability associated with H and then allocated the remaining to V and IH according to the endpoint probabilities of these phenotypes in SC-1. Following this approach, we simulated a family of curves that differ for initial conditions (see “Initial conditions” subsection in Material and Methods).

**Fig 3:**
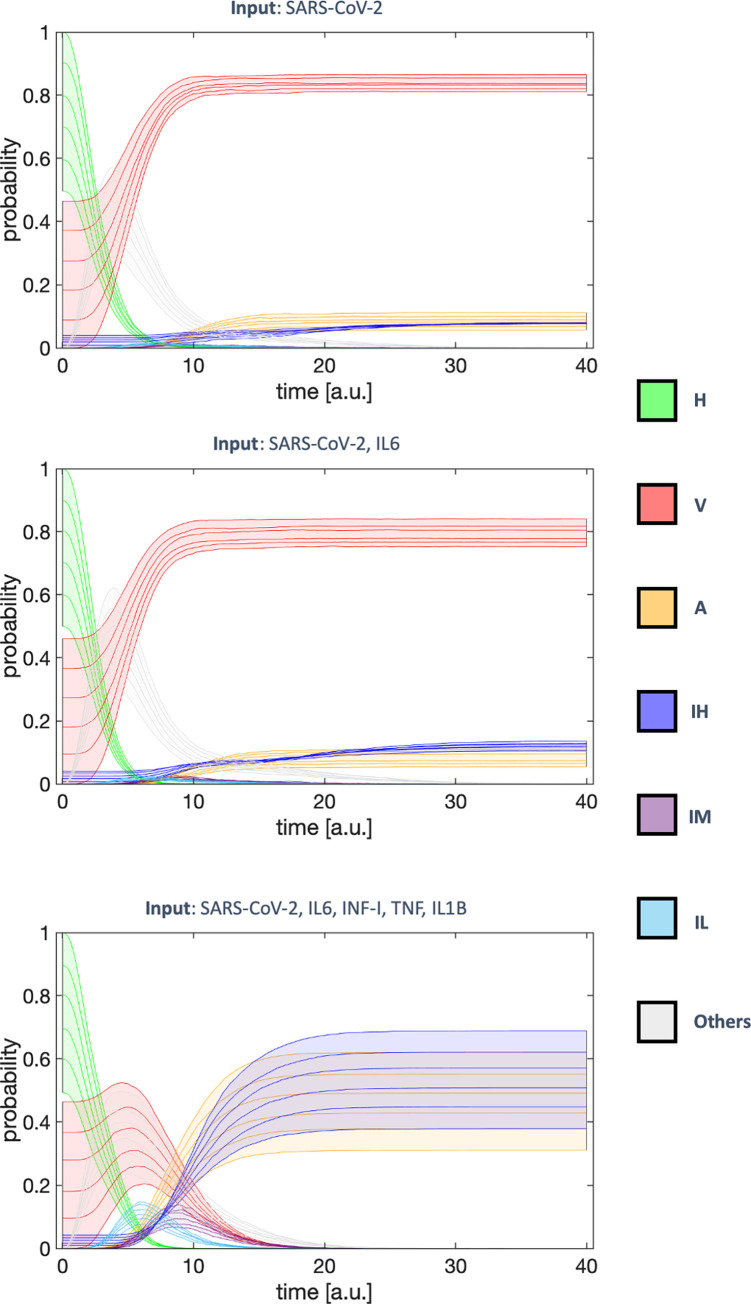
Dynamical evolution of the cell’s phenotype in SC-1(left), SC-2(center) and SC-3 (right). A heterogeneous epithelium is simulated in the different extracellular conditions (performed with MaBoSS [37]). The initial populations are a mix of H, V and IH. They are generated by defining the probability of H and then allocating the remaining proportionally to the endpoint probability of the first scenario. Each area represents a family of curves derived from the simulation at fixed initial conditions.

In **Fig 3** are reported the probability dynamics for the major phenotypes. While in the SC-1 and SC-2 we observe a quick increase in the number of infected cells (V) that becomes the dominant phenotype, in SC-3 we observe the disappearance of the V phenotype, and the system ends either in A or IH independently of the initial conditions. In this case, however, the transitory behavior of the system acquires particular importance. As a matter of fact, the V phenotype first increases reaching a peak which is a function of the initial condition, and then disappears progressively. Mild Inflammation phenotypes (IM and IL) transiently appear before becoming IH.

### Effect of drug treatment

The logical model presented in the previous sections has been used to test the effects of different treatments. For each drug, we searched for the mechanisms of action (MoA), selecting the corresponding nodes on the regulatory network and accordingly modulating the variable values (with a knock-down) along the simulation (**Table 2**). For this reason, we ignored drugs with unknown MoA, and focused on drugs that have been examined in human studies and that have an *in vitro* activity against either the viral replication (usually referred to the early phase disease) or the inflammatory reaction (second disease phase).

**Table 2:** Drug therapies and their efficacy scores. Drug therapies were simulated with their molecular targets and the drug-specific scores computed for each scenario. The overall score is the sum of the previous scores and serves as a general performance indicator. The scoring formula is explained in Materials and Methods. Each score in the specific scenarios is from -4 to 4 and considers the advantages of the cell in terms of enrichment of the favorable phenotypes or reduction of the unfavorable ones. The overall score has a minimum and a maximum, respectively, of -12 and 12. Drugs are sorted according to the overall score to highlight those that are predicted to perform better.

**Drugs**	**Targets**	**SC-1**	**SC-2**	**SC-3**	**Overall**
**Dexamethasone/Baricitinib/Remdesivir**	NfKb, JAK_IFNR, JAK_IL6R, Viral_dsRNA	3,98	3,98	3,33	11,29
**Dexamethasone/Remdesivir**	NfKb, Viral_dsRNA	2,66	1,74	2,72	7,13
**Remdesivir/Baricitinib**	JAK_IFNR, JAK_IL6R, Viral_dsRNA	2,37	2,73	1,53	6,63
**Remdesivir**	Viral_dsRNA	1,67	1	1,28	3,95
**Infliximab**	TNFt, TNF	0,28	0,33	0,31	0,92
**Dexamethasone/Baricitinib**	NfKb, JAK_IFNR, JAK_IL6R	-0,07	-0,06	0,9	0,77
**Dexamethasone**	NfKb	-0,03	-0,07	0,71	0,6
**Colchicine**	NLRP3	-0,04	-0,05	0,35	0,25
**Baricitinib**	JAK_IFNR, JAK_IL6R	-0,02	-0,04	0,13	0,07
**Tocilizumab**	IL6R	-0,01	-0,05	0,12	0,05
**Anakinra**	IL1bt, IL1b	0,03	-0,04	0,02	0,01

More specifically, Remdesivir is a SARS-CoV-2 RNA-dependent RNA polymerase inhibitor (model target: Viral_dsRNA), which inhibits viral replication. The clinical benefit of this treatment among hospitalized patients has been investigated in randomized trials with conflicting results [38–41]. Dexamethasone reduces inflammation by production of specific mediators and suppression of neutrophil migration (model target: NfKb). Data from randomized trials strongly support the role of glucocorticoids for severe COVID-19 [42,43]. Baricitinib is a Janus kinase (JAK) inhibitor with potent immunomodulatory effects and a potential antiviral role, blocking the viral entry in human cells (model target: JAK_IFNR and JAK_IL6R). Emerging data suggest that Baricitinib may provide a benefit for selected patients with severe disease in terms of reduced mortality. Currently, the combination of Dexamethasone and Baricitinib seems to be one of the most promising options for severe COVID-19 treatment [44,45]. IL-6 is one of the main inflammatory markers involved in the critical phase of COVID-19. Tocilizumab, an IL-6 blocker (model target: IL6R), has been associated with lower mortality in high quality studies [46–53]. Other agents that target other proinflammatory cytokines, like Anakinra (model target: IL1bt and IL1b) and Infliximab (model target: TNFt and TNF), have been investigated with uncertain results [54–56]. Colchicine has several potential mechanisms of action, including reducing the chemotaxis of neutrophils, inhibiting inflammasome signaling, and decreasing the production of cytokines [57] (model target: NLRP3). Although there exists some data demonstrating a benefit from the use of colchicine in early, mild to moderate COVID-19, the benefit is modest, there is no reduction in mortality, and adverse effects are common [13,58].

The therapies are simulated as a continuous administration of the drug (or combination therapy). Given the qualitative nature of the model, we simulated the system without side effects and guaranteeing full efficacy of the drugs (always in the therapeutic window).

We tested the drugs in the three previously described scenarios to evaluate their effects in reducing the emergence of disease-related phenotypes (*i*.*e*., all the phenotypes except H) with respect to the untreated model. The model does not account for a spatial description of the infection, but only for a local state of the tissue. We assumed that is the environment in which cells are embedded and its stimuli to define how they will respond; this allows to ideally connect the studied scenarios to disease stages with the appropriate chariness. A quantitative score has been defined to ease the comparison (see Materials and Methods) and has been calculated singularly, for each scenario, and globally, summing the three individual scores (**Table 2**). A non-aggregated table of scores with the singular score for each phenotype, scenario and treatment is reported in **Table B in S1 File**. The aim is to support in *silico* therapy selection based on the simulated scenarios, possibly related to the states of the disease severity, by providing a cumulative score as a quantitative indicator to evaluate lines of treatment.

In the model simulations, the effects of single drugs are limited and, in some cases, counterproductive. The anti-inflammatory drugs alone (*i*.*e*., Dexamethasone, Baricitinib, Tocilizumab [59] or Colchicine) exhibit a very mild effect against inflammation. In the first two scenarios, they appear counterproductive (negative score value), and they appear to produce more benefit when administered in a strong inflammatory scenario (**Fig A panel C-F in S1 File**). As reported in the work of Hubner *et al*. [60], Anakinra can reduce the inflammation in tumor cells co-cultured with PBMCs, but in the context of an inflammatory milieu it will increase the apoptotic rate. This can be also observed in our model simulation (**Fig A panel G in S1 File**), where the drug shows a positive effect on the reduction of IH but induces an increase of the A phenotype (**Table B in S1 File**).

Anti-TNF-α drugs, such as Infliximab, act as a mild anti-inflammatory and have a well-demonstrated ability to reduce levels of the cytokines associated with poor COVID-19 prognosis [61,62]. In our third simulation scenario, SC-3, the drug limits cell apoptosis allowing for stable viral proliferation. In SC-1 and SC-2, instead, Infliximab has a milder effect given the low number of inflamed cells (**Fig A panel H in S1 File**).

According to our simulations, the antiviral Remdesivir is the best single treatment for the early phase of the illness. Remdesivir acts by limiting viral replication when administered in the early stages of the disease, while it increases and accelerates a strong inflammatory reaction the more severe the disease scenario (**Fig A panel B in S1 File**).

We decided to explore also the potentialities of combination therapies by combining more than one class of drugs (**Fig B in S1 File**). The combinations of a single anti-inflammatory drug with antiviral treatment resulted as the second and third treatment in terms of efficacy. Dexamethasone plus Remdesivir reduce the overall inflammation and strongly inhibit cell infection. The combination of Baricitinib and Remdesivir, which shows in our simulations a good response especially in the first two scenarios, has been approved in patients who cannot receive corticosteroids [63]. In the following section, we have deeply analyzed these combinations. The only combination resulting in a low score is the one combining the two anti-inflammatory drugs, due to the increased probability of the apoptotic phenotype.

Finally, an interesting possibility underlined by the model is the triple combination of Remdesivir with Baricitinib and the Dexamethasone, which resulted in the treatment with the highest score. This novel combination will be analyzed in more detail in the next sections.

### Analysis of drug combinations between Baricitinib and Remdesivir

The combination of Baricitinib and Remdesivir in the trial ACCT2 was associated with a better prognosis in hospitalized patients with COVID-19 that do not require invasive ventilation, compared to the treatment with the antiviral alone [64]. No study comparing the effect of the combination therapy, or Baricinib alone, with placebo, has been performed due to compassion rule. Model simulations can be a valuable tool for investigating the therapy effect trying to unravel the phenomena underlying the treatments.

**Fig 4:**
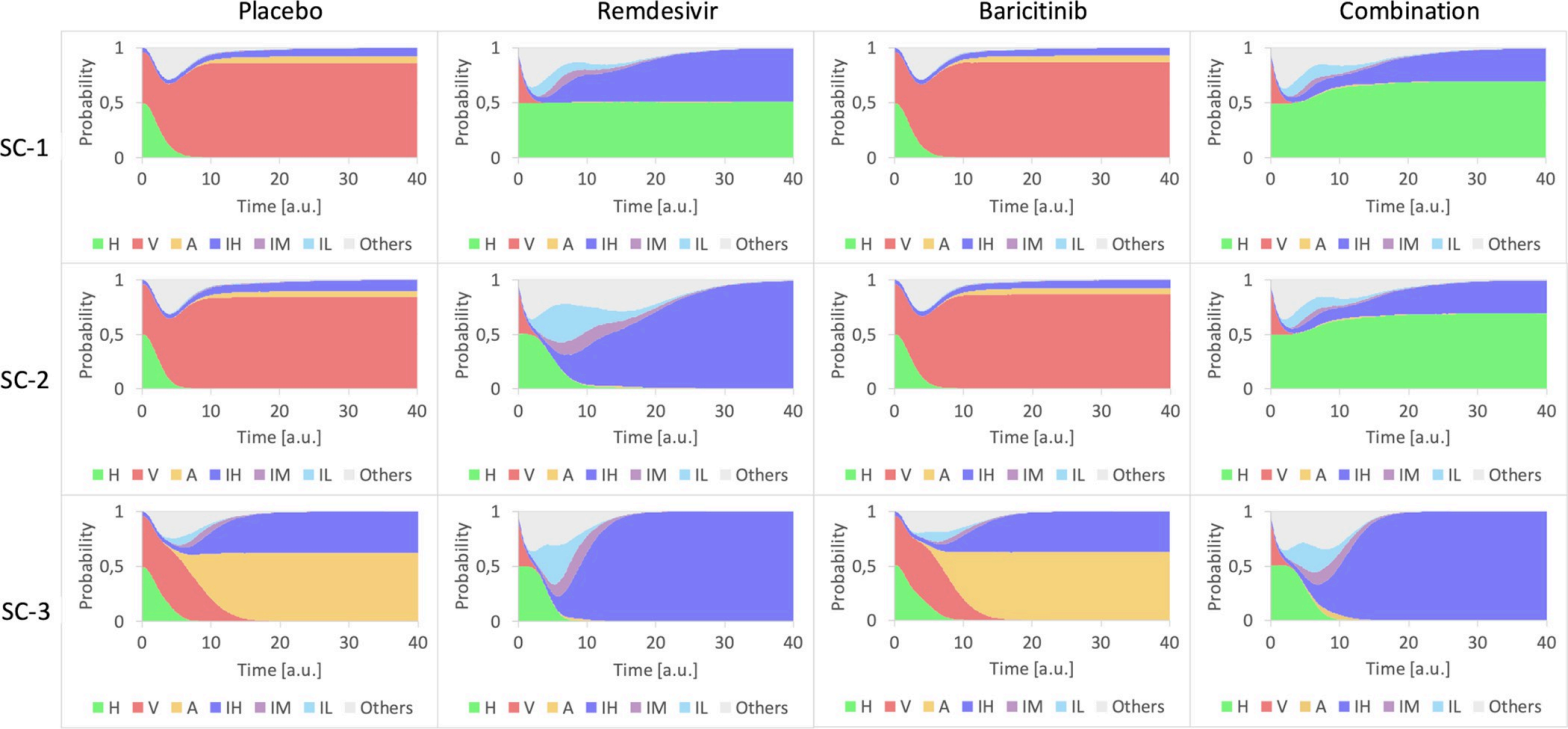
Temporal dynamics for Remdesivir, Baricitinib and their combination expressed as the probability to reach the most frequent phenotypes. Each row corresponds to a scenario, and each column to a treatment. The initial conditions correspond to 50% of healthy cells, and the remaining percentage proportionally distributed between the different untreated stable states (45.9% infected cells, 4.1% inflamed cells). The colored area represents the probability of each phenotype. Simulations were performed with MaBoSS [37].

The dynamics of the main stable phenotypes for placebo (here simulated as the untreated condition) are shown in **Fig 4** for the single therapy with Remdesivir or Baricitinib and their combination treatment, in the three previously described scenarios. The simulations of Remdesivir (**Fig 4**) highlight the importance of an early administration of the drug, as the first scenario is the one that maximizes the treatment benefit (maintaining H cells). However, this is because the new cell infection is blocked, while the infected cells evolve towards an IH. In the other scenarios, IH becomes dominant, the faster the evolution, the more severe the scenario. The treatment with Baricitinib alone has no major effect (**Fig 4**); besides a slight improvement by reducing the IH phenotype in SC-1 and SC-2.

Better results are obtained with the dual combination therapy (**Fig 4**). In mild (SC-1) and middle (SC-2) scenarios, the healthy population increases by reducing the population of inflamed cells, making the combination better than Remdesivir alone. The harsher scenario (SC-3) results similar to the antiviral therapy. To be noted, there is a time window during the switch between healthy and inflamed cells where the inflamed population is under control. This behavior could be considered in designing treatment regimens.

Since the combination therapy of Baricitinib and Remdesivir shows interesting additive effects, we used our mathematical representation of the cell to investigate the network circuits, trying to better understand the rationale behind the efficacy of combination therapies.

**Fig 5:**
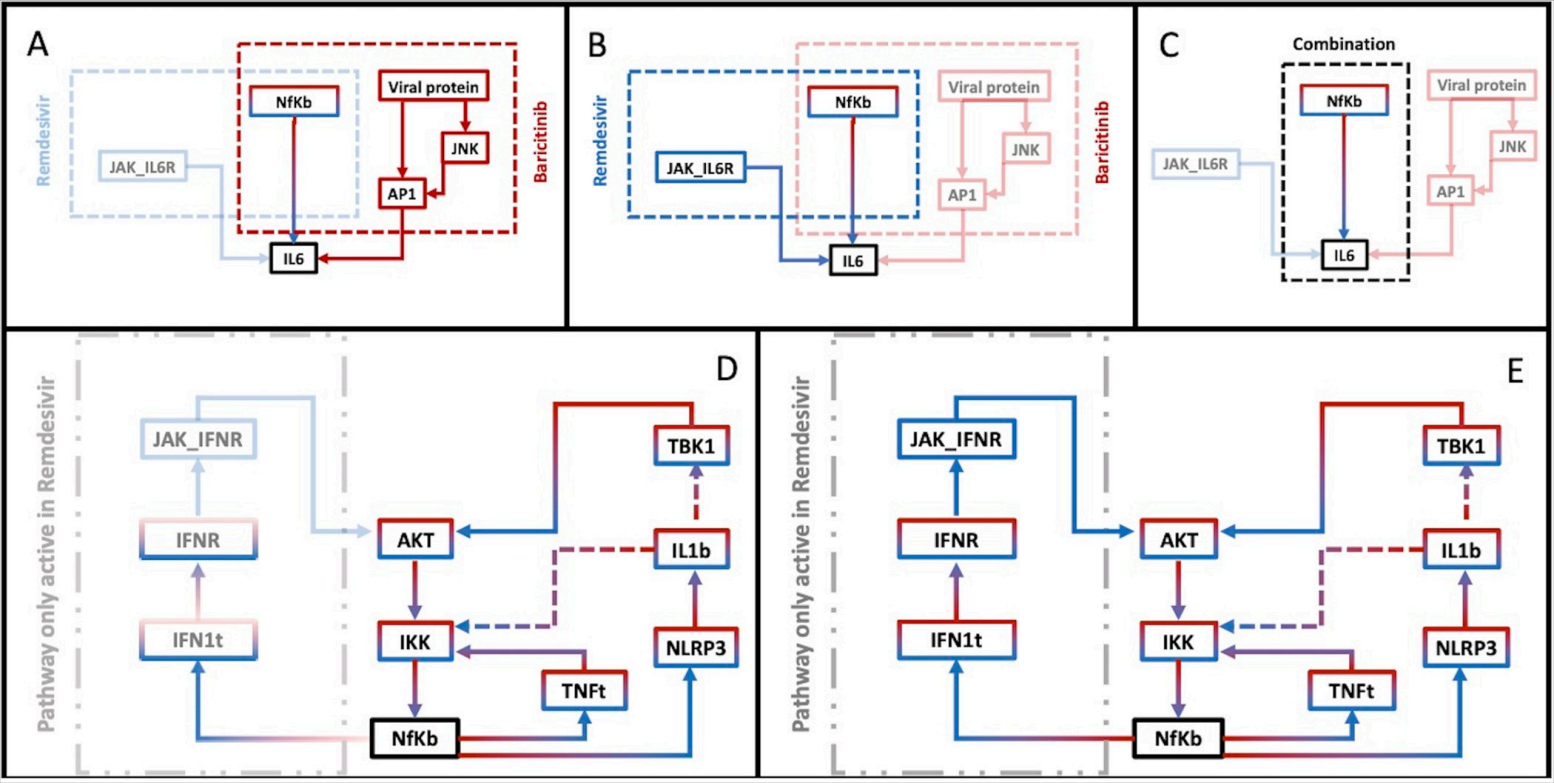
IL6 recruitment and NfKb activation. (A-C) The main regulatory pathways of IL6 obtained by reducing the regulatory network and propagating the fix values. Nodes and arrows are colored corresponding to the treatment studied. (A) IL6 is regulated in Baricitinib by NfKb and AP1 (red lines); (B) Remdesivir acts on IL6 through JAK_IL6R and NfKb (blue lines); (C) The combination therapy maintains only the IL6 regulation via NfKb (red-blue lines). (D-E) Regulatory pathways of NfKb for Remdesivir (blue), Baricitinib (red) and their combination (red-blue). (D) All pathways are activated for Remdesivir. JAK_IFNR is a specific node on a linear regulatory loop on NfKb, present only in Remdesivir. (E) For Baricinib and its combination with Remdesivir, the regulatory loops are reduced to three: a loop through TNF, and two loops via the Inflammasome and IL1b.

To highlight the reason for the additive effect of the cocktail of Baricitinib and Remdesivir, we reduced the regulatory graph as outlined in the Materials and Methods. The reduced graphs for each treatment have been analyzed and compared with each other. In terms of nodes, the combination graph appears to be the intersection between the two single drug graphs. In **Fig 5A and 5C** a detail of the regulation of IL-6 in the second scenario is reported (scenario corresponding to the second line in **Fig 4**). IL-6 is an important cytokine involved in a wide variety of biological functions such as inflammatory processes and immune recruitment. In Baricitinib, **Fig 5A**, IL-6 is positively regulated by NfKb and AP1, stimulated by the recognition of the viral proteins. In Remdesivir, **Fig 5B**, IL-6 is activated by NfKb or by its receptor via JAK. Both single therapies act by inhibiting just one of the three possible IL6 promotion processes. The combination therapy, instead, shows the additive effect of the two drugs resulting in a reduction of activation routes of IL-6, leaving only the NfKb route. In this case, NfKb acquires a fundamental role for the inflammation regulation being itself an inflammation promoter and an upstream regulator of IL-6. The fact that a path for IL-6 stimulation is still present might explain why the combination is not resolutive in the third scenario. This analysis can suggest NfKb as a good target for a concomitant or a subsequent treatment.

Due to the importance acquired by the NfKb node, we report in **Fig 5D and 5E** the positive feedback loop acting on NfKb. **Fig 5D** shows all the circuits in Remdesivir treatment, each one stimulated by endogenous production of IFN-I, TNF, and Inflammasome (IL-1β). In combination treatment and in Baricitinib alone, the circuit sustained by IFN-I is interrupted, reducing the feedback loops from four to three (two of them depending on the Inflammasome action).

**Fig 6:**
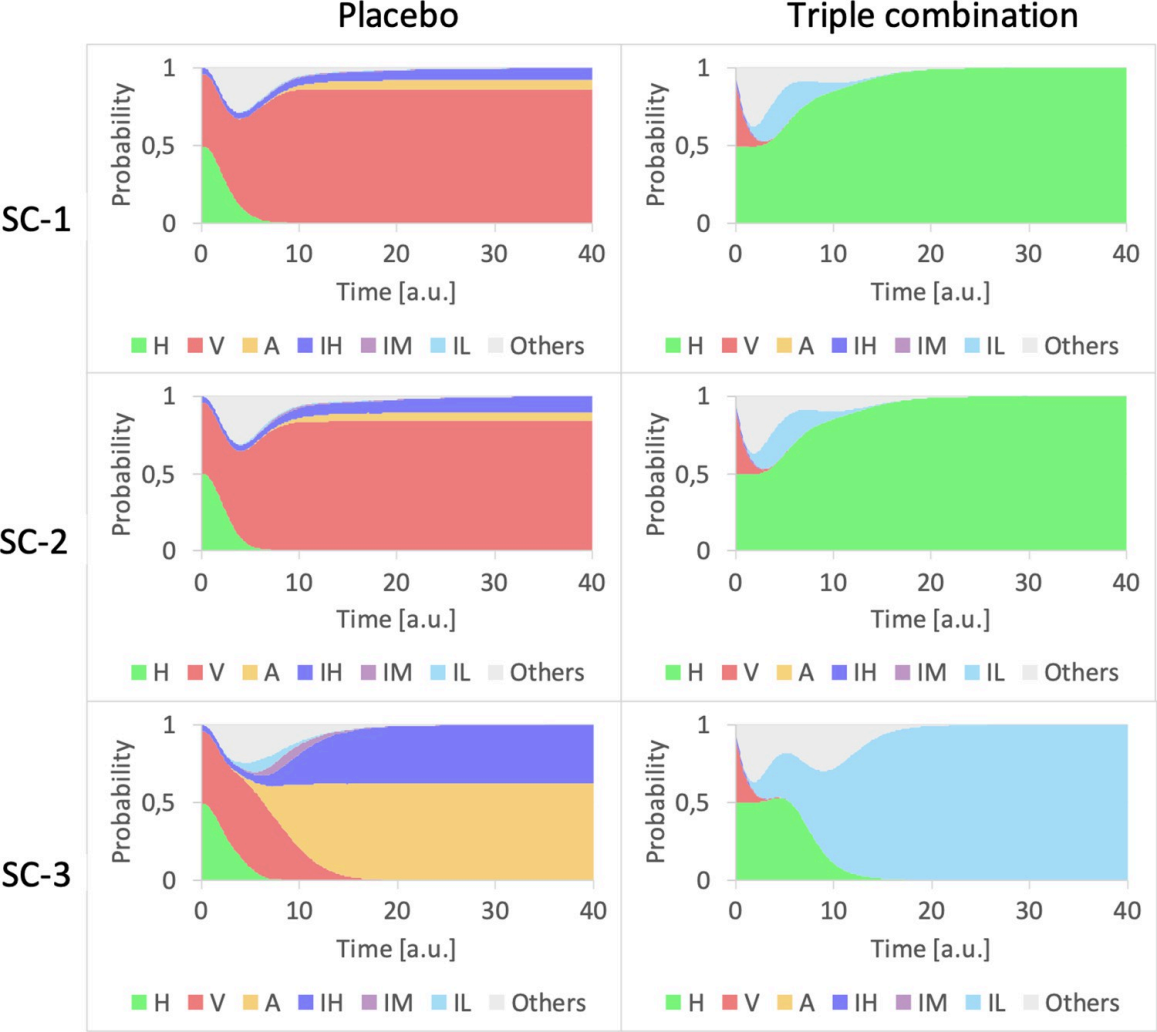
Triple combination compared with placebo. The first column represents the placebo treatment whereas the second column the triple combination of Remdesivir, Baricitinib, and Corticosteroid, administered together. The rows correspond to the different scenarios. The colored areas represent the cumulative probability to measure a certain percentage of phenotypes for each biopsy. Simulations were performed with MaBoSS [37].

The previous analysis of the double combination together with the pivotal role of NFkB as inflammation regulator [65], make it an ideal target for treatment. Here, we analyze the effects of a triple combination of Remdesivir, Baricitinib and Dexamethasone (which affects NfKb), in the three scenarios (**Fig 6**). In SC-1 and SC-2, the triple combination appears completely effective. For an analysis of the effects of the reduced efficacy of the drugs, see **Fig C in S1 File**. In the worst-case scenario, the triple combination can control the inflammation response that remains at a low level and inhibits viral replication. The temporal dynamics of the phenotypes are quite similar in the three scenarios. The triple combination is at this moment studied in the AMMURAVID clinical trial [66].

## Discussion/Conclusion

From the beginning of the COVID-19 pandemic, researchers from every scientific field have joined forces to face the new global challenge. The present model aims to study the complex network of molecular interactions following viral infection of an epithelial cell (*e*.*g*., a lung epithelial cell). The logical approach compensates for the lack of quantitative data (*e*.*g*., interaction rates, biochemical parameters, species concentrations etc.), allowing to reproduce and study complex networks.

The logical model was built based on the molecular mechanisms describing the viral entry and the consequent immune system and inflammatory response. We started considering only the initial cellular hallmarks of the COVID-19 disease, leaving the advanced hallmarks involving tissue responses (*e*.*g*., fibrosis, ER stress, stress granules) to future extensions of the model involving multiple compartments. Various extracellular inputs have been considered allowing us to explore a wide range of scenarios of cellular infection. Inputs were set as external stimuli coming from the close environment (e.g. virions, cytokines, chemokines or stress signals). However, we decided to focus on the most compelling disease scenarios, thus leaving the complete exploration of the input importance to future work. In addition to the scenarios considered for drug repurposing, the model can also simulate the consequences of possible bacterial infections resulting from the onset of COVID-19 disease, stimuli due to growth factors, and anti-inflammatory immune stimuli. The model can be instantiated in different stages of the disease depicting the infection spreading among cells with high viral load, the resolution of the disease, returning to physiological normal levels, or its evolution into an intense and uncontrolled inflammatory state characterized almost by an absence of viral replication. This last case represents the most fatal and dangerous scenario for the host as well as the most difficult to deal with from a therapeutic point of view. Given its focus on the single cell, our simulation approach can characterize only the localized response of the tissue and cannot be reproduced at a multicellular/tissue/organism level. The modeling scenarios therefore do not coincide necessarily with the clinical stages of the illness but are connected to them if we consider them restricted to limited areas of tissue (*e*.*g*., the lung tissue) or as a mean field approximation. The choice of the modeling technique, of the pathways introduced as well as the initial conditions imposed limited interpretations of the results, which, although reproducing various biological behaviors, remain qualitative, spatially local and temporally limited (not involving many systemic secondary regulatory mechanisms).

The model drug simulations agree with the major findings regarding antiviral and immune-therapy for COVID-19 [59]. In our scenario, each drug is considered to have a full efficacy at the target level and no side effects. Analysis of simulations with a reduced drug efficacy revealed that no new phenotypes (other than untreated/placebo) emerged, and that the drug effects are proportional to their effectiveness rate (**Fig C in S1 File**).

Simulation results suggested that the effects of single drugs are limited, or sometimes counterproductive. Anti-inflammatory drugs alone have a mild effect also in contrasting the cytokine storm. Anakinra reduces inflammation at the cost of an increase in apoptotic tissue. In its most severe scenario, Infliximab reduces the apoptotic population preserving a replication niche. The antiviral, Remdesivir appears the most effective treatment in the early stages of the disease but less useful, if not counterproductive, in case of severe inflammation.

We also simulated combinations for initial stages (SC-1), intermediate evolution of the disease (SC-2), and cytokine storm (SC-3). The combination of an anti-inflammatory drug (Dexamethasone or Baricitinib) with the anti-viral Remdesivir is effective in all the stages of the illness. In particular, the treatment with Baricitinib and Remdesivir shows additive effects of the two drugs. To identify and disentangle the combinatorial effects, we proceeded by analyzing the model structure. In particular, the combination reduces the activation pathways of IL6 (an important inflammation promoter) to a single path involving NFkB (**Fig 5**). Baricitinib, moreover, acts also indirectly over NFkB, blocking the activation chain by IFN-I and its receptor. Therefore, NFkB assumes a crucial role in the immune system regulation suggesting this protein complex as the perfect target for a further combination. Finally, the model does not account for the toxicity of the drugs, making it important to take into further consideration cost and benefit ratio when the protocol is identified.

From the model analysis, Baricitinib and the Dexamethasone have different molecular targets, and that they have complementary anti-inflammatory actions on the system. For these reasons, a triple combination (*i*.*e*., Remdesivir, Baricitinib and the Dexamethasone) can be envisaged, which might accelerate the recovery, reducing the inflammation and blocking the viral progression. The model provides insights on the mechanism of action underlying this combination, further supporting the ongoing clinical trial [66].

The model presents different temporal dynamics, which have to be considered qualitative indications of the existence of a time window that could be exploited for therapeutic purposes. It is however necessary to experimentally verify its duration. In this work, because of the difficulties in obtaining the biological data necessary for calibration and the complexity of the regulatory graph, all rates have been considered unitary, so the time should be interpreted as a semiquantitative variable. A possible extension of the model could calibrate the rates from the experimental data. The single-cell focus of the model limits its application to the understanding of the molecular mechanisms, or the localized phenomena underlying the disease. Possible extensions of this work could leverage on the current model, after proper simplification, and define a minimal set of nodes to be embedded in a multicellular environment such as that defined by programs as Epilog [67] or Physicell [68].

*In silico* models can be instrumental to explore the complex networks and pathways that define a biological process. The understanding of the phenomena and their characterization, together with the identification of new drug targets and the test of drug combinations, can become pivotal in the fight against epidemic outbreaks. Logical models, or other parameters-free approaches, are the sharpest tools to use in this fight since they can be developed and instantiated with just qualitative knowledge, thus guiding the process of research while other information is collected. Here we presented an example of such an approach, by developing a logical model of the COVID-19 infection and testing the effect of possible treatments.

## Materials and methods

### Model construction

The model construction steps are schematically summarized as follows:

1. Starting from the Covid-19 hallmarks list defined by SIGNOR database we first selected those that describe early events of the infection process (*e*.*g*., virus-entry, activation of the innate immune system, inflammation, apoptosis, MAPK activation) and excluded the pathways activated at late stages or involving phenomena out of the study target (*e*.*g*., fibrosis, ER stress, stress granules).
2. We analyzed the SIGNOR network for each hallmark considered. The interaction rules were carefully revised and included in our regulatory network, only the molecules involved in a regulatory cascade belonging to the hallmarks were considered. A particular attention was devoted to minimize the network size using the minimal number of nodes necessary to describe a particular cascade or multistep process (*e*.*g*., the binding of the spike protein to the ACE2 receptor and the following mechanistic processes are resume in a single node).
3. Finally, the input nodes considered in our model were the external nodes that regulate the activation pathways of our readouts (the input signals secreted by around cells).

### Computational tools and methods

The COVID-19 logical model represented in **Fig 1** was built using GINsim [69] (version 3.0.0b, http://ginsim.org/). This software is dedicated to logical formalism and includes different functionalities such as: determination of the stable states, export facilities, reachability analysis, etc. In asynchronous update, model attractors are associated to reachability probabilities, which can be estimated using a built-in GINsim functionality implementing Avatar [36], a modified Monte Carlo simulation, with number of runs set to 10^4^, expansion limit and rewiring limit 100, ensuring the convergence of estimated probabilities.

More quantitative view of the dynamics is provided by stochastic simulations as performed by MaBoSS (https://maboss.curie.fr/ [37]). The COVID-19 model was exported into MaBoSS format using GINsim features. MaBoSS computes stochastic trajectories and provides the time evolution of probabilities of the component values. We considered equal transition rates, a time step of 0.1, and a simulation time of 40 with 10^4^ runs.

Model reduction was applied when necessary, using the GINsim built-in function. This process allows the user to remove the selected components while maintaining a consistent dynamical behavior. For each hidden component the logical rules associated with the targets are redefined to account for the (indirect) effects of its regulators. The reduction used in the current work can be found saved in the GINsim model file (**S5 File**).

### Initial conditions

A family of simulations (**Fig 3**) has been defined for each scenario and the system initialized to the states H, V and IH following the equations:

$$0.5\leq prob(H)\leq 1prob(V)=\frac{probFinSC1(V)\cdot 100}{probFinSC1(V)+probFinSC1(IH)}=\frac{81\cdot 100}{88}(1-prob(H))=92\%(1-prob(H))$$

$$prob(IH)=\frac{probFinSC1(IH)\cdot 100}{probFinSC1(V)+probFinSC1(IH)}=\frac{7\cdot 100}{88}(1-prob(H))=8\%(1-prob(H))$$

where *prob*(*i*), with *i* = *H*, *V*, *IH*, is the initial probability of the state *i*; *probFinSC*1(*j*), with *j* = *V*, *IH*, is the final state probability for the SC-1 obtained with GINsim, using Avatar simulation as explained in the methods, simulating the system starting from the H state.

The drugs effect was instead simulated, in the three different scenarios, by setting the model initial conditions to 50% of healthy cells, and the remaining percentage proportionally distributed between the different untreated stable states (45.9% infected cells, 4.1% inflamed cells).

### Drug scores

We defined an efficiency score to facilitate the comprehension and allow the comparison between the drug treatments. The overall score is the sum of the single total scores of the different scenarios and can be used to compare one drug with the others in a qualitative way.

We used the phenotypes probability dynamics to compute the areas under the phenotype curves (AUC) for all the treated and untreated cases, for the two initial conditions, *i* = *α*, *β*, corresponding to 100% (*α*) and 50% (*β*) of initial healthy cells.

The healthy Raw Score (*RS*_*H*_) has been computed as in Formula (1), mediating the difference between healthy AUC for drug and placebo, for the two initial conditions. *RS*_*H*_ is positive when the drug induces a temporal increase in the number of healthy cells.


$${RS}_{H}=\frac{1}{2}[\left({AUC}_{drug,H,\alpha }-{AUC}_{placebo,H,\alpha })+({AUC}_{drug,H,\beta }-{AUC}_{placebo,H,\beta }\right)]$$ (1)

The viral and apoptotic raw scores (*RS*_*U*_ with *U* = *V*, *A*) have been computed as in Formula (1) but inverting the terms of the differences in order to have a positive score in case of reduction of the populations, see Eq (2).


$${RS}_{U}=\frac{1}{2}[\left({AUC}_{placebo,U,\alpha }-{AUC}_{drug,U,\alpha })+({AUC}_{placebo,U,\beta }-{AUC}_{drug,U,\beta }\right)]$$ (2)

The inflamed raw score *RS*_*I*_, instead, has been computed scaling the three different inflammation classes AUC with their inflammation level and then mediated as in the previous Eq (2). Defining as in Eq (3) the total contribution of the three classes (High HI, Medium MI, Low LI Inflammation) described in **Table 1**, the score *RS*_*I*_ has been computed as in Eq (4).


$${AUC}_{i}=3∙{AUC}_{HI,i}+{2∙AUC}_{MI,i}+{AUC}_{LI,i}$$ (3)

$${RS}_{I}=\frac{1}{2}[\left({AUC}_{placebo,U,\alpha }-{AUC}_{drug,U,\alpha })+({AUC}_{placebo,U,\beta }-{AUC}_{drug,U,\beta }\right)]$$ (4)

Finally, each raw score *RS*_*j*_ with *j* = *H*, *V*, *A*, *I* has been normalized over the maximum for each scenario when the raw score was positive. If the raw score was negative, it has been normalized over the minimum of each scenario and changed sign. In the end, the final score for each scenario has been obtained normalizing over the maximum in absolute value. The final score *S* = ∑*S*_*j*_ for each scenario ranges between -4 and 4 and it is positive when the drug showed an improvement compared to placebo. The overall score represents the sum of the final scores S for the three scenarios and ranges between -12 and 12.

## Supporting information

S1 File Supplementary sections and figures. (PDF)

S2 File Table providing model node annotations. (XLSX)

S3 File Table providing model interaction annotations. (XLSX)

S4 File Table providing the model stable states. (XLSX)

S5 File Annotated model as GINsim file and as SMBL-qual. (ZIP)

## References

[1] Cherry JD. The chronology of the 2002–2003 SARS mini pandemic. 2004; 262–269. doi: 10.1016/j.prrv.2004.07.009 15531249 PMC7106085

[2] Banerjee A, Baid K, Mossman K. Molecular Pathogenesis of Middle East Respiratory Syndrome (MERS) Coronavirus. 2019; 139–147. doi: 10.1007/s40588-019-00122-7 32226718 PMC7100557

[3] Widagdo W, Okba NMA, Raj VS, Haagmans BL. MERS-coronavirus: From discovery to intervention. One Heal. 2017;3: 11–16. doi: 10.1016/j.onehlt.2016.12.001 28616497 PMC5454172

[4] Ganesh B, Rajakumar T, Malathi M. Epidemiology and pathobiology of SARS-CoV-2 (COVID-19) in comparison with SARS, MERS: An updated overview of current knowledge and future perspectives. Clin Epidemiol Glob Heal. 2021;10: 100694. doi: 10.1016/j.cegh.2020.100694 33462564 PMC7806455

[5] Goddard AF, Patel M. SARS-CoV-2 variants and ending the COVID-19 pandemic. 2021;397: 952–954. doi: 10.1016/S0140-6736(21)00370-6 33581803 PMC7906631

[6] This C, Cent ACS, Li Y, Tenchov R, Liu C, Watkins S. A Comprehensive Review of the Global Efforts on COVID-19 Vaccine Development. 2021;2. doi: 10.1021/acscentsci.1c00120 34056083 PMC8029445

[7] Harrison AG, Lin T, Wang P. Mechanisms of SARS-CoV-2 Transmission and Pathogenesis. Trends Immunol. 2020;41: 1100–1115. doi: 10.1016/j.it.2020.10.004 33132005 PMC7556779

[8] Paules CI, Fauci AS. COVID-19: The therapeutic landscape. Med. 2021;2: 493–497. doi: 10.1016/j.medj.2021.04.015 33899041 PMC8057546

[9] Sette A, Crotty S. ll Adaptive immunity to SARS-CoV-2 and COVID-19. Cell. 2021;184: 861–880. doi: 10.1016/j.cell.2021.01.007 33497610 PMC7803150

[10] Blanco-Melo D, Nilsson-Payant BE, Liu WC, Uhl S, Hoagland D, Møller R, et al. Imbalanced Host Response to SARS-CoV-2 Drives Development of COVID-19. Cell. 2020;181: 1036–1045.e9. doi: 10.1016/j.cell.2020.04.026 32416070 PMC7227586

[11] Chen R, Lan Z, Ye J, Pang L, Liu Y, Wu W, et al. Cytokine Storm: The Primary Determinant for the Pathophysiological Evolution of COVID-19 Deterioration. Frontiers in Immunology. Front Immunol; 2021. doi: 10.3389/fimmu.2021.589095 33995341 PMC8115911

[12] Siddiqi HK, Mehra MR. COVID-19 illness in native and immunosuppressed states: A clinical–therapeutic staging proposal. Journal of Heart and Lung Transplantation. 2020. pp. 405–407. doi: 10.1016/j.healun.2020.03.012 32362390 PMC7118652

[13] Riva L, Yuan S, Yin X, Martin-Sancho L, Matsunaga N, Pache L, et al. Discovery of SARS-CoV-2 antiviral drugs through large-scale compound repurposing. Nature. 2020;586. doi: 10.1038/s41586-020-2577-1 32707573 PMC7603405

[14] Wang X. MINIREVIEWS COVID—19 drug repurposing: A review of computational screening methods, clinical trials, and protein interaction assays. 2020; 1–24. doi: 10.1002/med.21728 32864815 PMC8049524

[15] Shah B, Modi P, Sagar SR. In silico studies on therapeutic agents for COVID-19: Drug repurposing approach. Life Sci. 2020;252: 117652. doi: 10.1016/j.lfs.2020.117652 32278693 PMC7194845

[16] Zhou Y, Wang F, Tang J, Nussinov R, Cheng F. Review Artificial intelligence in COVID-19 drug repurposing. 2020;7500. 10.1016/S2589-7500(20)30192-8 PMC7500917 32984792

[17] Uttam T, Subhashree S, Madhu P, Lingaraju C, Kesavan M, Kumar D. Drug repurposing approach to fi ght COVID-19. 2020. 10.1007/s43440-020-00155-6 PMC7474498 32889701

[18] Xiang Y, Jia Y, Chen L, Guo L, Shu B. COVID-19 epidemic prediction and the impact of public health interventions: A review of COVID-19 epidemic models. Infect Dis Model. 2021;6: 324–342. doi: 10.1016/j.idm.2021.01.001 33437897 PMC7790451

[19] Kotanmi B, Gnanvi JE, Gl R. On the reliability of predictions on Covid-19 dynamics: A systematic and critical review of modelling techniques. 2021;6: 258–272. doi: 10.1016/j.idm.2020.12.008 33458453 PMC7802527

[20] Chen T, Philip M, Cao KL, Tyagi S. A multi-modal data harmonisation approach for discovery of COVID-19 drug targets. 2021;00: 1–15. doi: 10.1093/bib/bbab185 34036326 PMC8194516

[21] Nawaz MS, Abbas PF, Hamido S. Using artificial intelligence techniques for COVID-19 genome analysis. 2021; 3086–3103. doi: 10.1007/s10489-021-02193-w 34764587 PMC7888282

[22] Chen LYC, Hoiland RL, Stukas S, Wellington CL, Sekhon MS. Assessing the importance of interleukin-6 in COVID-19. The Lancet Respiratory Medicine. Lancet Publishing Group; 2021. p. e13. doi: 10.1016/S2213-2600(20)30600-7 33460569 PMC7836242

[23] Id ALJ, Id RAA, Id SA, Id VC, Deng X, Id APS, et al. COVID-19 virtual patient cohort suggests immune mechanisms driving disease outcomes. 2021. doi: 10.1371/journal.ppat.1009753 PMC8312984 34260666

[24] Soni B, Singh S. COVID-19 co-infection mathematical model as guided through signaling structural framework. Comput Struct Biotechnol J. 2021;19: 1672–1683. doi: 10.1016/j.csbj.2021.03.028 33815692 PMC7997053

[25] Tay MZ, Poh CM, Rénia L, MacAry PA, Ng LFP. The trinity of COVID-19: immunity, inflammation and intervention. Nature Reviews Immunology. 2020. pp. 363–374. doi: 10.1038/s41577-020-0311-8 32346093 PMC7187672

[26] Ostaszewski M, Mazein A, Gillespie ME, Kuperstein I, Niarakis A, Hermjakob H, et al. COVID-19 Disease Map, building a computational repository of SARS-CoV-2 virus-host interaction mechanisms. Scientific Data. Nature Research; 2020. doi: 10.1038/s41597-020-0477-8 32371892 PMC7200764

[27] Licata L, Lo Surdo P, Iannuccelli M, Palma A, Micarelli E, Perfetto L, et al. SIGNOR 2.0, the SIGnaling Network Open Resource 2.0: 2019 update. Nucleic Acids Res. 2020;48: D504–D510. doi: 10.1093/nar/gkz949 31665520 PMC7145695

[28] Abou-Jaoudé W, Traynard P, Monteiro PT, Saez-Rodriguez J, Helikar T, Thieffry D, et al. Logical Modeling and Dynamical Analysis of Cellular Networks. Front Genet. 2016;7: 94. doi: 10.3389/fgene.2016.00094 27303434 PMC4885885

[29] Vatansever HS, Becer E. Relationship between IL-6 and COVID-19: To be considered during treatment. Future Virology. Various; 2020. pp. 817–822. doi: 10.2217/fvl-2020-0168

[30] Xiao H, Xu LH, Yamada Y, Liu DX. Coronavirus spike protein inhibits host cell translation by interaction with eIF3f. PLoS One. 2008;3: e1494. doi: 10.1371/journal.pone.0001494 18231581 PMC2204050

[31] Conti P, Ronconi G, Caraffa A, Gallenga CE, Ross R, Frydas I, et al. Induction of pro-inflammatory cytokines (IL-1 and IL-6) and lung inflammation by Coronavirus-19 (COVI-19 or SARS-CoV-2): anti-inflammatory strategies. Journal of biological regulators and homeostatic agents. J Biol Regul Homeost Agents; 2020. pp. 327–331. doi: 10.23812/CONTI-E 32171193

[32] Wilson JG, Simpson LJ, Ferreira AM, Rustagi A, Roque J, Asuni A, et al. Cytokine profile in plasma of severe COVID-19 does not differ from ARDS and sepsis. JCI Insight. 2020;5. doi: 10.1172/jci.insight.140289 32706339 PMC7526438

[33] Dinarello CA. Interleukin-1 in the pathogenesis and treatment of inflammatory diseases. Blood. 2011;117: 3720–3732. doi: 10.1182/blood-2010-07-273417 21304099 PMC3083294

[34] Ye Q, Wang B, Mao J. The pathogenesis and treatment of the ‘Cytokine Storm” in COVID-19.’ Journal of Infection. J Infect; 2020. pp. 607–613. doi: 10.1016/j.jinf.2020.03.037 32283152 PMC7194613

[35] Chen J, Qi T, Liu L, Ling Y, Qian Z, Li T, et al. Clinical progression of patients with COVID-19 in Shanghai, China. J Infect. 2020;80: e1–e6. doi: 10.1016/j.jinf.2020.03.004 32171869 PMC7102530

[36] Mendes ND, Monteiro PT, Carneiro J, Remy E, Chaouiya C. Quantification of reachable attractors in asynchronous discrete dynamics. 2014. Available from: http://arxiv.org/abs/1411.3539.

[37] Stoll G, Viara E, Barillot E, Calzone L. Continuous time boolean modeling for biological signaling: application of Gillespie algorithm. BMC Syst Biol. 2012;6: 116. doi: 10.1186/1752-0509-6-116 22932419 PMC3517402

[38] Repurposed Antiviral Drugs for Covid-19—Interim WHO Solidarity Trial Results. N Engl J Med. 2021;384: 497–511. doi: 10.1056/NEJMoa2023184 33264556 PMC7727327

[39] Siemieniuk RAC, Bartoszko JJ, Ge L, Zeraatkar D, Izcovich A, Pardo-Hernandez H, et al. Drug treatments for covid-19: Living systematic review and network meta-Analysis. BMJ. 2020;370: 28. doi: 10.1136/bmj.m2980 32732190 PMC7390912

[40] Wang Y, Zhang D, Du G, Du R, Zhao J, Jin Y, et al. Remdesivir in adults with severe COVID-19: a randomised, double-blind, placebo-controlled, multicentre trial. Lancet. 2020;395: 1569–1578. doi: 10.1016/S0140-6736(20)31022-9 32423584 PMC7190303

[41] Ansems K, Grundeis F, Dahms K, Mikolajewska A, Thieme V, Piechotta V, et al. Remdesivir for the treatment of COVID-19. Cochrane Database Syst Rev. 2021;2021. doi: 10.1002/14651858.CD014962 34350582 PMC8406992

[42] Sterne JAC, Murthy S, Diaz J V., Slutsky AS, Villar J, Angus DC, et al. Association between Administration of Systemic Corticosteroids and Mortality among Critically Ill Patients with COVID-19: A Meta-analysis. JAMA—J Am Med Assoc. 2020;324: 1330–1341. doi: 10.1001/jama.2020.17023 32876694 PMC7489434

[43] Wagner C, Griesel M, Mikolajewska A, Mueller A, Nothacker M, Kley K, et al. Systemic corticosteroids for the treatment of COVID-19. Cochrane Database Syst Rev. 2021;2021. doi: 10.1002/14651858.CD014963 34396514 PMC8406706

[44] Kalil AC, Patterson TF, Mehta AK, Tomashek KM, Wolfe CR, Ghazaryan V, et al. Baricitinib plus Remdesivir for Hospitalized Adults with Covid-19. N Engl J Med. 2021;384: 795–807. doi: 10.1056/NEJMoa2031994 33306283 PMC7745180

[45] Marconi VC, Ramanan A V., de Bono S, Kartman CE, Krishnan V, Liao R, et al. Efficacy and safety of baricitinib for the treatment of hospitalised adults with COVID-19 (COV-BARRIER): a randomised, double-blind, parallel-group, placebo-controlled phase 3 trial. Lancet Respir Med. 2021;9: 1407–1418. doi: 10.1016/S2213-2600(21)00331-3 34480861 PMC8409066

[46] Group TWREA for C-19 T (REACT) W, Domingo P, Mur I, Mateo GM, Gutierrez M del M, Pomar V, et al. Association Between Administration of IL-6 Antagonists and Mortality Among Patients Hospitalized for COVID-19: A Meta-analysis. JAMA. 2021;326: 499–518. doi: 10.1001/jama.2021.11330 34228774 PMC8261689

[47] Abani O, Abbas A, Abbas F, Abbas M, Abbasi S, Abbass H, et al. Tocilizumab in patients admitted to hospital with COVID-19 (RECOVERY): a randomised, controlled, open-label, platform trial. Lancet (London, England). 2021;397: 1637–1645. doi: 10.1016/S0140-6736(21)00676-0 33933206 PMC8084355

[48] Rosas IO, Bräu N, Waters M, Go RC, Hunter BD, Bhagani S, et al. Tocilizumab in Hospitalized Patients with Severe Covid-19 Pneumonia. N Engl J Med. 2021;384: 1503–1516. doi: 10.1056/NEJMoa2028700 33631066 PMC7953459

[49] Stone JH, Frigault MJ, Serling-Boyd NJ, Fernandes AD, Harvey L, Foulkes AS, et al. Efficacy of Tocilizumab in Patients Hospitalized with Covid-19. N Engl J Med. 2020;383: 2333–2344. doi: 10.1056/NEJMoa2028836 33085857 PMC7646626

[50] Hermine O, Mariette X, Tharaux P-L, Resche-Rigon M, Porcher R, Ravaud P, et al. Effect of Tocilizumab vs Usual Care in Adults Hospitalized With COVID-19 and Moderate or Severe Pneumonia: A Randomized Clinical Trial. JAMA Intern Med. 2021;181: 32–40. doi: 10.1001/jamainternmed.2020.6820 33080017 PMC7577198

[51] Salvarani C, Dolci G, Massari M, Merlo DF, Cavuto S, Savoldi L, et al. Effect of Tocilizumab vs Standard Care on Clinical Worsening in Patients Hospitalized with COVID-19 Pneumonia: A Randomized Clinical Trial. JAMA Intern Med. 2021;181: 24–31. doi: 10.1001/jamainternmed.2020.6615 33080005 PMC7577199

[52] Salama C, Han J, Yau L, Reiss WG, Kramer B, Neidhart JD, et al. Tocilizumab in Patients Hospitalized with Covid-19 Pneumonia. N Engl J Med. 2021;384: 20–30. doi: 10.1056/NEJMoa2030340 33332779 PMC7781101

[53] Mariette X, Hermine O, Tharaux PL, Resche-Rigon M, Steg PG, Porcher R, et al. Effectiveness of Tocilizumab in Patients Hospitalized with COVID-19: A Follow-up of the CORIMUNO-TOCI-1 Randomized Clinical Trial. JAMA Internal Medicine. American Medical Association; 2021. pp. 1241–1243. doi: 10.1001/jamainternmed.2021.2209 34028504 PMC8145157

[54] Kyriazopoulou E, Poulakou G, Milionis H, Metallidis S, Adamis G, Tsiakos K, et al. Early treatment of COVID-19 with anakinra guided by soluble urokinase plasminogen receptor plasma levels: a double-blind, randomized controlled phase 3 trial. Nat Med. 2021;27: 1752–1760. doi: 10.1038/s41591-021-01499-z 34480127 PMC8516650

[55] Mariette X, Hermine O, Resche-Rigon M, Porcher R, Ravaud P, Bureau S, et al. Effect of anakinra versus usual care in adults in hospital with COVID-19 and mild-to-moderate pneumonia (CORIMUNO-ANA-1): a randomised controlled trial. Lancet Respir Med. 2021;9: 295–304. doi: 10.1016/S2213-2600(20)30556-7 33493450 PMC7825875

[56] Fisher BA, Veenith T, Slade D, Gaskell C, Rowland M, Whitehouse T, et al. Namilumab or infliximab compared with standard of care in hospitalised patients with COVID-19 (CATALYST): a randomised, multicentre, multi-arm, multistage, open-label, adaptive, phase 2, proof-of-concept trial. Lancet Respir Med. 2022;10: 255–266. doi: 10.1016/S2213-2600(21)00460-4 34922649 PMC8676420

[57] Reyes AZ, Hu KA, Teperman J, Wampler Muskardin TL, Tardif JC, Shah B, et al. Anti-inflammatory therapy for COVID-19 infection: The case for colchicine. Annals of the Rheumatic Diseases. Ann Rheum Dis; 2021. pp. 550–557. doi: 10.1136/annrheumdis-2020-219174 33293273 PMC8491433

[58] Tardif JC, Bouabdallaoui N, L’Allier PL, Gaudet D, Shah B, Pillinger MH, et al. Colchicine for community-treated patients with COVID-19 (COLCORONA): a phase 3, randomised, double-blinded, adaptive, placebo-controlled, multicentre trial. Lancet Respir Med. 2021;9: 924–932. doi: 10.1016/S2213-2600(21)00222-8 34051877 PMC8159193

[59] Cantini F, Goletti D, Petrone L, Najafi Fard S, Niccoli L, Foti R. Immune Therapy, or Antiviral Therapy, or Both for COVID-19: A Systematic Review. Drugs. Adis; 2020. pp. 1929–1946. doi: 10.1007/s40265-020-01421-w 33068263 PMC7568461

[60] Hübner M, Effinger D, Wu T, Strauß G, Pogoda K, Kreth FW, et al. The IL-1 antagonist anakinra attenuates glioblastoma aggressiveness by dampening tumor-associated inflammation. Cancers (Basel). 2020;12. doi: 10.3390/cancers12020433 32069807 PMC7072290

[61] Robinson PC, Liew DFL, Liew JW, Monaco C, Richards D, Shivakumar S, et al. The Potential for Repurposing Anti-TNF as a Therapy for the Treatment of COVID-19. Med. Elsevier BV; 2020. pp. 90–102. doi: 10.1016/j.medj.2020.11.005 33294881 PMC7713589

[62] Feldmann M, Maini RN, Woody JN, Holgate ST, Winter G, Rowland M, et al. Trials of anti-tumour necrosis factor therapy for COVID-19 are urgently needed. The Lancet. Lancet Publishing Group; 2020. pp. 1407–1409. doi: 10.1016/S0140-6736(20)30858-8 32278362 PMC7158940

[63] FDA. Baricitinib Letter of Authorization Revised July 28 2021. In: FDA website [Internet]. 2021. Available: https://www.fda.gov/media/143822/download.

[64] Marconi VC, Palacios GMR, Hsieh L, Kline S, Tapson V, Iovine NM, et al. Baricitinib plus Remdesivir for Hospitalized Adults with Covid-19. 2019; 1–13. doi: 10.1056/NEJMoa2031994 PMC7745180 33306283

[65] Tripathi P, Aggarwal A. NF-kB transcription factor: a key playe génération of immune response. 2006;90: 519–531.

[66] NCT04832880. Factorial Randomized Trial of Rendesivir and Baricitinib Plus Dexamethasone for COVID-19 (the AMMURAVID Trial). 2021. Available from: https://clinicaltrials.gov/ct2/show/NCT04832880.

[67] Varela PL, Ramos C V., Monteiro PT, Chaouiya C. EpiLog: A software for the logical modelling of epithelial dynamics. F1000Research. 2018;7: 1145. doi: 10.12688/f1000research.15613.2 30363398 PMC6173114

[68] Ghaffarizadeh A, Heiland R, Friedman SH, Mumenthaler SM, Macklin P. PhysiCell: An open source physics-based cell simulator for 3-D multicellular systems. PLoS Comput Biol. 2018;14: e1005991. doi: 10.1371/journal.pcbi.1005991 29474446 PMC5841829

[69] Naldi A, Hernandez C, Abou-Jaoudé W, Monteiro PT, Chaouiya C, Thieffry D. Logical Modeling and Analysis of Cellular Regulatory Networks With GINsim 3.0. Front Physiol. 2018;9: 646. doi: 10.3389/fphys.2018.00646 29971008 PMC6018412

