# Supplementary material for: A novel logical model of COVID-19 intracellular infection to support therapies development
Source: PLoS Comput Biol. 2022 Aug 29;18(8):e1010443. doi: 10.1371/journal.pcbi.1010443 (PMC9462742; doi:10.1371/journal.pcbi.1010443)
Supplement: S1 File — (PDF) [file pcbi.1010443.s001.pdf]

A novel logical model of COVID-19 intracellular infection to support therapies development

Elena Piretto, Gianluca Selvaggio, Damiano Bragantini, Enrico Domenici, Luca Marchetti

S1 file: Supplementary sections and figures

Drug simulations: dynamical evolution of the cell's phonotype

The dynamical evolution of the phenotypes has been studied for all the drugs tested in the main text and compared with the untreated case.

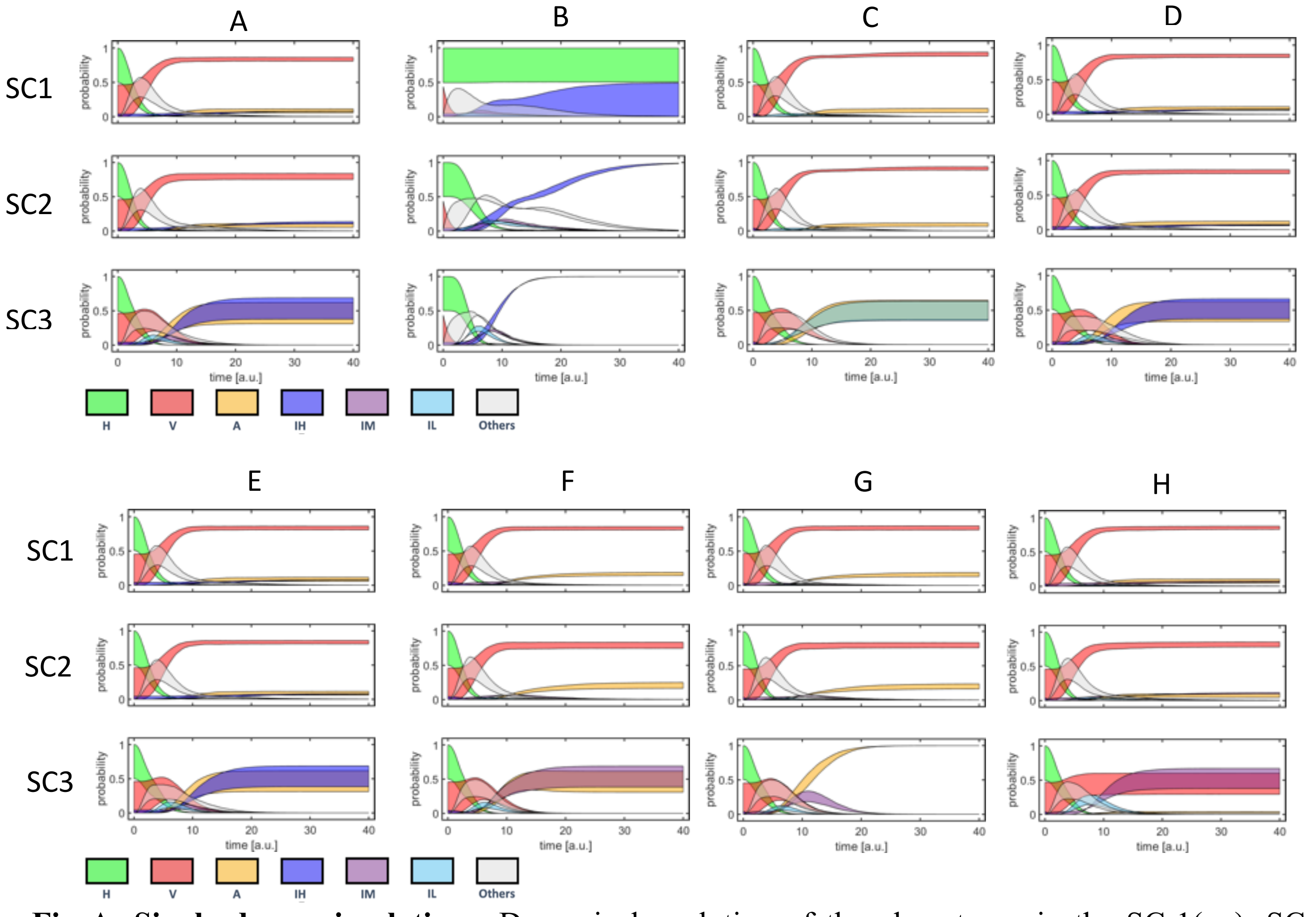

Fig A: **Single drugs simulations.** Dynamical evolution of the phenotypes in the SC-1(up), SC-2(center) and SC-3 (down). The initial populations are a mix of H, V and IH. They are generated by defining the probability of H and then allocating the remaining proportionally to the endpoint probability of the first scenario. Each area represents a family of curves derived from the simulation at a fixed initial condition. All the drugs have been administered at the starting point as mentioned in the main text. A) Untreated population; B) Remdesivir; C) Dexamethasone; D) Baricitinib; E) Tocilizumab; F) Colchicine; G) Anakinra; H) Infliximab.

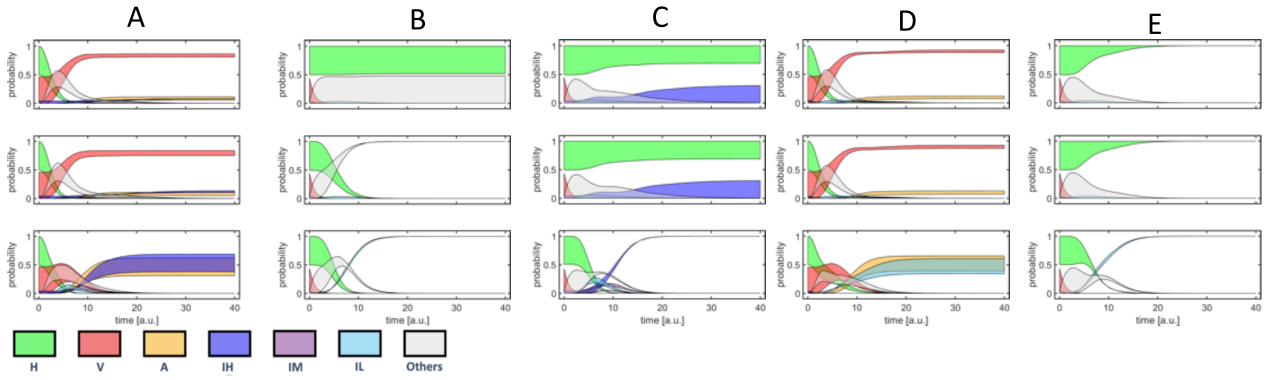

Fig B: **Combination therapy simulations.** Dynamical evolution of the phenotypes in the SC-1(up), SC-2(center) and SC-3 (down). The initial populations are a mix of H, V and IH. They are generated by defining the probability of H and then allocating the remaining proportionally to the endpoint probability of the first scenario. Each area represents a family of curves derived from the simulation at a fixed initial condition. All the drugs have been administered at the starting point as mentioned in the main text and simultaneously. A) Untreated population; B) Dexamethasone with Remdesivir; C) Baricitinib with Remdesivir; D) Dexamethasone with Baricitinib; E) Dexamethasone with Remdesivir and Baricitinib.

Drug reduced efficacy

In our simulations, for simplicity, we considered all the drugs always completely effective against their targets. In the reality, each drug has a reduced level of efficacy depending on many factors. Here we investigate for Remdesivir the effects of reduced efficacy. More in detail, computationally, the percentage of success of the drug has been reduced to 95% or 80% and the resulting phenotypes analyzed. From a comparison between the full-efficacy results with the partial ones, the phenotypes that emerged were the untreated-case phenotypes. Also, the abundance of the phenotypes has been redistributed in the percentage of unsuccess with the same probability of the untreated case.

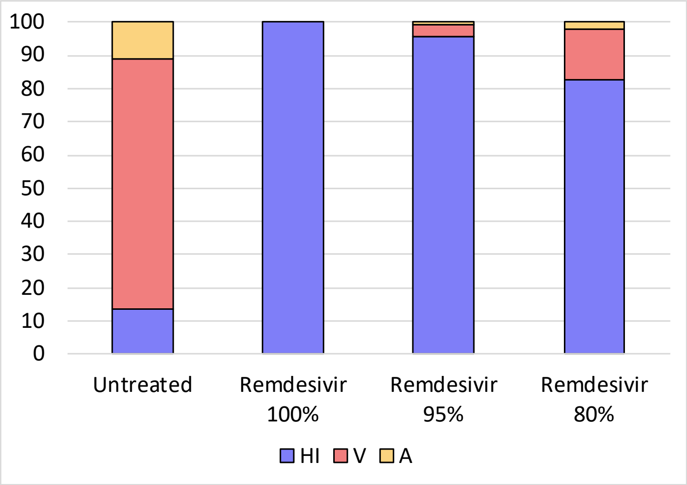

Fig C: Reduced drug efficacy. The probability to obtain the final phenotypes in the untreated case (*i.e.,* HI, V, A) is compared with the probability for Remdesivir with different levels of efficacy (100%, 95% and 80%).

Stable States and phenotypic repertoire

Several input combinations can lead to the same phenotypes depending on the complete initial state. From this point of view, it can be useful to consider both, the percentage of states for each output combination (see table 1) that can permit defining the amplitude of the attraction basin and, the biological meaning since several states can be computational artefacts.

Inputs											Outputs				phenotypes
SARS_CoV_2	IL1B_e	AGT_e	TGFb_e	IFNI_e	IL6_e	TNF_e	EGF_e	IL10_e	PAMPs	FASL	Infected	Apoptosis	Immune Response	Inflammation	
0	0	0	0	0	0	0	0	0	0	0	0	0	0	0	
1	*	0	*	1	*	0	0	1	0	0	0	0	0	0	H
0	*	0	*	*	*	0	0	1	0	0	0	0	0	0	
1	*	0	*	1	*	0	1	1	0	0	0	0	0	1	
0	*	0	*	*	*	0	1	1	0	0	0	0	0	1	
*	*	0	*	*	*	*	0	1	1	*	0	0	1	0	
*	*	1	*	*	*	0	0	1	0	0	0	0	1	0	
*	*	1	*	*	*	*	0	1	1	*	0	0	1	0	IL
*	*	0	*	*	*	*	1	1	1	*	0	0	1	1	
*	*	1	*	*	*	0	1	1	0	0	0	0	1	1	
*	*	1	*	*	*	*	1	1	1	*	0	0	1	1	
*	*	*	*	*	*	*	*	0	*	*	0	0	2	3	IH
0	0	0	0	0	0	0	0	0	0	1	0	1	0	0	
1	*	0	1	1	*	*	0	1	0	*	0	1	0	0	
1	*	0	0	1	*	0	0	1	0	1	0	1	0	0	
1	*	0	0	1	*	1	0	1	0	0	0	1	0	0	
1	*	0	0	1	*	1	0	1	0	1	0	1	0	0	
0	*	0	1	*	*	*	0	1	0	*	0	1	0	0	
0	*	0	0	*	*	0	0	1	0	1	0	1	0	0	
0	*	0	0	*	*	1	0	1	0	0	0	1	0	0	
0	*	0	0	*	*	1	0	1	0	1	0	1	0	0	A
1	*	0	*	1	*	*	1	1	0	*	0	1	0	1	
0	*	0	*	*	*	*	1	1	0	*	0	1	0	1	
*	*	1	1	*	*	*	0	1	0	*	0	1	1	0	
*	*	1	0	*	*	0	0	1	0	1	0	1	1	0	
*	*	1	0	*	*	1	0	1	0	0	0	1	1	0	
*	*	1	0	*	*	1	0	1	0	1	0	1	1	0	
*	*	1	*	*	*	*	1	1	0	*	0	1	1	1	
*	*	*	*	*	*	*	*	*	*	*	1	1	0	1	
*	*	*	*	*	*	0	*	*	*	0	1	0	0	1	V

Table A: Phenotypic repertoire with their inputs. Table of the phenotypes shown in Table1 with their inputs. "*" means that their values can be either 0 or 1.

Non-aggregated Scores

A quantitative score has been defined to ease the comparison (see Materials and Methods) and has been calculated singularly, for each scenario, for each treatment (single drug or combination). The singular raw scores for each phenotype are reported in Table B. Each raw score has a value between 1 (if the effect is positive, i.e., it increases the healthy phenotype or reduce the dangerous one) and -1 (if the effect is negative).

SC-1	RSH	RSI	RSV	RSA	sum
Dexamethasone/Baricitinib/Remdesivir	1,00	0,98	1,00	1,00	3,98
Remdesivir/Baricitinib	0,80	-0,42	1,00	0,99	2,37
Dexamethasone/Remdesivir	0,69	-0,03	1,00	1,00	2,66
Remdesivir	0,68	-1,00	1,00	0,99	1,67
Dexamethasone/Baricitinib	0,00	1,00	-0,91	-0,16	-0,07
Dexamethasone	0,00	1,00	-1,00	-0,03	-0,03
Baricitinib	0,00	0,11	-0,13	0,00	-0,02
Tocilizumab	0,00	0,00	0,00	-0,02	-0,01
Colchicine	0,00	0,95	0,00	-1,00	-0,04
Anakinra	0,00	0,96	-0,02	-0,92	0,03
Infliximab	0,00	0,41	-0,23	0,11	0,28
SC-2	RSH	RSI	RSV	RSA	sum
Dexamethasone/Baricitinib/Remdesivir	1,00	0,98	1,00	1,00	3,98
Remdesivir/Baricitinib	0,80	-0,06	1,00	0,99	2,73
Dexamethasone/Remdesivir	0,02	-0,28	1,00	1,00	1,74
Remdesivir	0,02	-1,00	1,00	0,98	1,00
Dexamethasone/Baricitinib	0,00	1,00	-0,91	-0,15	-0,06
Dexamethasone	0,00	0,99	-1,00	-0,06	-0,07
Baricitinib	0,00	0,40	-0,39	-0,05	-0,04
Tocilizumab	0,00	0,35	-0,35	-0,05	-0,05
Colchicine	0,00	0,94	0,01	-1,00	-0,05
Anakinra	0,00	0,95	-0,03	-0,96	-0,04
Infliximab	0,00	0,39	-0,19	0,14	0,33
SC-3	RSH	RSI	RSV	RSA	sum
Dexamethasone/Baricitinib/Remdesivir	1,00	0,33	1,00	1,00	3,33
Remdesivir/Baricitinib	0,51	-0,97	1,00	0,99	1,53
Dexamethasone/Remdesivir	0,42	0,30	1,00	1,00	2,72
Remdesivir	0,28	-1,00	1,00	1,00	1,28
Dexamethasone/Baricitinib	0,29	0,73	0,00	-0,12	0,90
Dexamethasone	0,08	0,71	-0,01	-0,08	0,71
Baricitinib	0,11	0,04	0,02	-0,04	0,13
Tocilizumab	0,09	0,00	0,03	0,00	0,12
Colchicine	0,01	0,33	0,01	0,00	0,35
Anakinra	0,02	1,00	0,00	-1,00	0,02
Infliximab	0,03	0,35	-1,00	0,93	0,31

Table B: Non-aggregated scores. Table of the scores for each scenario and each treatment considered. RSH is the healthy Raw Score, RSI is the inflamed raw score, RSV is the infected raw score, RSA is the apoptotic raw score (see Material and Methods).
